# Visceral Adiposity Index Plays an Important Role in Prognostic Prediction in Patients With Non-ST-Segment Elevation Acute Coronary Syndrome and Type 2 Diabetes Mellitus Undergoing Percutaneous Coronary Intervention

**DOI:** 10.3389/fcvm.2021.735637

**Published:** 2021-11-18

**Authors:** Qi Zhao, Yu-Jing Cheng, Ying-Kai Xu, Zi-Wei Zhao, Chi Liu, Tie-Nan Sun, Yu-Jie Zhou

**Affiliations:** Clinical Center for Coronary Heart Disease, Beijing Key Laboratory of Precision Medicine of Coronary Atherosclerotic Disease, Department of Cardiology, Beijing Anzhen Hospital, Beijing Institute of Heart Lung and Blood Vessel Disease, Capital Medical University, Beijing, China

**Keywords:** visceral adiposity index, insulin resistance, type 2 diabetes mellitus, non-ST-segment elevation acute coronary syndrome, percutaneous coronary intervention, prognosis

## Abstract

**Background:** Visceral adiposity index (VAI), a surrogate marker of adiposity and insulin resistance, has been demonstrated to be significantly related to cardiovascular disease. It remains indistinct whether VAI predicts adverse prognosis after percutaneous coronary intervention (PCI) for patients with non-ST-segment elevation acute coronary syndrome (NSTE-ACS) and type 2 diabetes mellitus (T2DM).

**Methods:** A total of 798 participants who met the enrollment criteria were finally brought into this study. VAI was determined by waist circumference, body mass index, fasting triglyceride, and high-density lipoprotein cholesterol as previously reported. Adverse prognosis included all-cause death, non-fatal myocardial infarction, non-fatal ischemic stroke, and ischemia-driven revascularization, the composite of which was defined as the primary endpoint.

**Results:** Higher VAI maintained as a significant and independent risk predictor for the primary endpoint, regardless of the adjustment for the various multivariate models [hazard ratio (95% CI) for fully adjusted model: 2.72 (2.02–3.68), *p* < 0.001]. The predictive value of VAI was further confirmed in sensitivity analysis where VAI was taken as a continuous variate. There was a dose-response relationship of VAI with the risk of the primary endpoint (*p* for overall association < 0.001). Moreover, the ability of VAI on the prediction of the primary endpoint was consistent between subgroups stratified by potential confounding factors (all *p* for interaction > 0.05). VAI exhibited a significant incremental effect on risk stratification for the primary endpoint beyond existing risk scores, expressed as increased Harrell's C-index, significant continuous net reclassification improvement, and significant integrated discrimination improvement.

**Conclusion:** VAI is a significant indicator for predicting worse prognosis and plays an important role in risk stratification among patients with NSTE-ACS and T2DM undergoing elective PCI. The present findings require further large-scale, prospective studies to confirm.

## Introduction

Coronary artery disease (CAD) has become one of the most important health issues over the years in China (1). Despite sufficient attention and intervention having been settled in clinical practice, the cardiovascular risk for patients with CAD, particularly for those who experienced acute coronary syndrome (ACS) and coupled with type 2 diabetes mellitus (T2DM), remains noteworthy (2, 3). T2DM, occurring in more than two-thirds of patients with ACS, has been widely demonstrated to be significantly related to the occurrence, progression, and prognosis of ACS (4, 5), which appeals to great efforts on identification of the risk factors mediating the close relationship in this specific high-risk population of ACS accompanied with T2DM.

Insulin resistance (IR), the major pathogenesis of T2DM, was proved to be prominently associated with cardiovascular disease (6, 7). Former studies have shown that IR is usually characterized as glycometabolic abnormality, lipometabolic disturbance, and visceral obesity (8). Given these characteristics, a calculated surrogate parameter called visceral adiposity index (VAI), which is determined by waist circumference (WC), body mass index (BMI), fasting triglyceride (TG), and high-density lipoprotein cholesterol (HDL-C), was proposed and shown to be highly related to the hyperinsulinemic-euglycemic (HIEG) clamp and the homeostasis model assessment of IR (HOMA-IR), the gold standard and frequently used methods for assessing IR (9, 10). Previous studies have demonstrated that there is a positive association between the level of VAI and the risk of atherosclerosis (11, 12). Moreover, former studies also revealed that a higher level of VAI was remarkably related to increased incidence and severity of cardiovascular disease (13–19).

At present, the potential of VAI in the prediction of worse prognosis after percutaneous coronary intervention (PCI) in patients with non-ST-segment elevation acute coronary syndrome (NSTE-ACS) and T2DM is still unknown. Therefore, this study was designed to explore the underlying relationship of VAI with long-term prognosis in this selected high-risk group and determine whether VAI is superior to existing risk scores on risk stratification.

## Materials and Methods

### Study Population

As a single-center observational cohort study, we retrospectively screened patients who underwent elective PCI in the Beijing Anzhen Hospital, Capital Medical University from January to December 2015. Patients diagnosed with NSTE-ACS [non-ST-segment elevation myocardial infarction (NSTEMI) or unstable angina (UA)] and coupled with a previous unequivocal diagnosis of T2DM or a newly diagnosed diabetes during hospitalization were enrolled in the study. Diagnostic criteria of NSTE-ACS and diabetes were referred to relevant guidelines, respectively (20, 21). Patients with definite or plausible type 1 diabetes mellitus, deficient data, and other exclusion criteria were excluded (details shown in Supplementary Figure 1). In total, 798 participants were ultimately brought into the current analysis.

### Data Collection and Definition

Demographic information, clinical characteristics, laboratory results, and medical and procedural therapeutic processes were acquired by referring to the electronic medical record management system of the Beijing Anzhen Hospital and then entered into an established database.

Body mass index was calculated as weight (kg)/[height (m)]^2^. WC was measured with a soft ruler at the end of exhalation and before the beginning of inspiration, defined as the horizontal girth through the center of the umbilical or the midpoint line between the inferior margin of the ribcage and the upper edge of the iliac crest. Patients who have smoked ≥ 100 cigarettes or drunk ≥ 12 times over the past year were considered to have a history of smoking or drinking, respectively. Patients with at least one first-degree family member having CAD were considered to have a family history of CAD. Patients with hypertension were defined as those having systolic blood pressure (SBP)/diastolic blood pressure (DBP) ≥ 140/90 mm Hg more than two times on different days during hospitalization or previous diagnosis of hypertension with antihypertensive treatments. Previous medical histories of myocardial infarction (MI), PCI, stroke, and peripheral artery disease (PAD) were obtained from self-reported information and then confirmed by relevant medical records. Stroke included cerebral infarction and transient ischemic attack and PAD was defined as the artery disease that happened other than coronary arteries with stenosis ≥ 50% and associated ischemic symptoms and/or signs. Laboratory indices were examined with standard techniques at the core laboratory by using blood samples extracted on an ≥ 8 h fasting state. VAI was calculated as: [WC (cm)/(39.68 + 1.88 × BMI)] × [fasting TG (mmol/L)/1.03] × [1.31/HDL-C (mmol/L)] for men and [WC (cm)/(36.58 + 1.89 × BMI)] × [fasting TG (mmol/L)/0.81] × [1.52/HDL-C (mmol/L)] for women (22).

Coronary angiography data were judged by at least two experienced professionals who were blinded to the study protocol. The disease characteristics were defined with reference to related guidelines (23, 24). The synergy between PCI with taxus and cardiac surgery (SYNTAX) score, calculated by the tool on the website (www.syntaxscore.com), was used to evaluate the disease complexity. PCI was performed by referring to present guidelines in China (25) and the experience of the chief cardiologist. Complete revascularization was defined as successful interventional procedures (residual stenosis ≤ 20%) in all the coronary lesions with diameter ≥ 1.5 mm and stenosis ≥ 50%.

The Thrombolysis in Myocardial Infarction (TIMI) score for NSTE-ACS was calculated as previous study described (26). The Global Registry of Acute Coronary Events (GRACE) score was calculated by using the online risk calculator (www.gracescore.org/website/WebVersion.aspx).

### Study Endpoint

Each participant received routine postdischarge follow-up until the occurrence of death or up to 48 months. The prognostic data were identified by telephonic interview and further verified by analyzing relevant medical records if indistinct information was acquired. The study endpoint events included all-cause death, non-fatal MI, non-fatal ischemic stroke, and ischemia-driven revascularization, the composite of which was defined as the primary endpoint. The first endpoint event for each participant that happened during the follow-up period was selected for the current analyses. The death resulted from any causes was defined as all-cause death. MI and ischemic stroke were defined in accordance with relevant guidelines, respectively (27, 28). Ischemia-driven revascularization was defined as any target or non-target vessel revascularization, either PCI or surgical bypass, on account of myocardial ischemia judged by symptoms, ECGs, and/or images.

### Statistical Analysis

Data analyses were performed with the SPSS IBM Statistics (version 26.0) (SPSS Inc., Chicago, Illinois, USA) and the R Programming Language (version 3.6.3) (Auckland University, New Zealand). A *p*-value (two-tailed) < 0.05 suggested as statistical significance.

Continuous variates were described as mean ± SD or median with interquartile range (25 and 75%) and tested by the *t*-test or the Mann–Whitney *U*-test correspondingly. Nominal variates were described as numbers with percentages and tested by the chi-squared test (with or without continuity correction) or the Fisher's exact test accordingly.

The time-dependent cumulative incidences of adverse events between VAI median groups were analyzed by the Kaplan–Meier method and tested by log-rank test. Unadjusted and adjusted Cox regression analyses were performed to evaluate the value of VAI on the prediction of adverse prognosis. Five models (models 1–5) were established and variates of which were selected based on the results of unadjusted analysis (*p* < 0.05) and clinical significance. Variates with potential collinearity were not selected simultaneously. The final selected variates in each model were as follows: model 1: age and gender; model 2: model 1 and SBP, DBP, smoking history, duration of diabetes, previous MI, previous PCI, previous stroke, and diagnosis; model 3: model 2 and total cholesterol (TC), estimated glomerular filtration rate (eGFR), glycosylated hemoglobin A1c (HbA1c), and left ventricular ejection fraction (LVEF); model 4: model 3 and statins at admission, oral antidiabetic drugs (OADs) at admission, insulin at admission, and angiotensin-converting enzyme inhibitor (ACEI)/angiotensin receptor blocker (ARB) at discharge; and model 5: model 4 and the SYNTAX score, left main artery (LM) treatment, complete revascularization, and number of drug-eluting stent (DES). VAI was analyzed as a nominal variate in the primary analysis and then as a continuous variate in the sensitivity analysis. The results were expressed as hazard ratio (HR) and 95% CI. The dose-response relationship of VAI with the risk of the primary endpoint was illustrated by restricted cubic smoothing adjusted for model 5. Further subgroup analysis according to various potential confounders was used to demonstrate the robustness of VAI in predicting the primary endpoint with the adjustment for model 5. Variates applied for grouping were excluded, respectively, and the interaction between each subgroup was examined by the likelihood ratio test.

The Harrell's C-index, continuous net reclassification improvement (NRI), and integrated discrimination improvement (IDI) were analyzed to elucidate whether VAI exhibited stronger abilities on risk stratification for the primary endpoint compared with existing risk scores and to investigate the incremental effects of VAI on risk stratification for the primary endpoint beyond existing risk scores.

## Results

The mean age of the participants was 60.9 ± 8.3 years old and 68.3% was men. Over the follow-up, a total of 231 (28.9%, 87.7 events per 1,000 person-years) primary endpoint events were observed, which consisted of 17 (2.1%, 5.4 events per 1,000 person-years) all-cause death, 47 (5.9%, 15.5 events per 1,000 person-years) non-fatal MI, 18 (2.3%, 5.8 events per 1,000 person-years) non-fatal ischemic stroke, and 149 (18.7%, 53.5 events per 1,000 person-years) ischemia-driven revascularization.

### General Characteristics of the Study Participants

The study participants were split into two groups based on the median of VAI, general characteristics of which are given in Tables 1, 2. Lower age, lower proportion of males and drinking history, higher BMI, WC, heart rate, and higher incidence of hypertension were observed in those with higher VAI. In addition, more participants were diagnosed with NSTEMI in the higher VAI group. As for laboratory examinations, participants with higher VAI exhibited higher levels of TG, TC, high-sensitivity C-reactive protein (hs-CRP), uric acid, fasting blood glucose (FBG), and HbA1c, while lower level of HDL-C. In the higher VAI group, more participants were prescribed ACEI/ARB and β-blocker for treatment. Additionally, the TIMI score for NSTE-ACS was significantly higher in those with higher VAI. There were no significant differences between lower and higher VAI groups with respect to coronary angiographic and procedural data.

**Table 1:** Baseline demographic, clinical, and laboratory characteristics of the study population.

	**Total population (*n* = 798)**	**Lower VAI (<2.6; *n* = 399)**	**Higher VAI (≥2.6; *n* = 399)**	***P*-value**
Age, years	60.9 ± 8.3	62.2 ± 7.7	59.6 ± 8.7	<0.001
Gender, male, *n* (%)	545 (68.3)	311 (77.9)	234 (58.6)	<0.001
BMI, kg/m^2^	26.7 ± 3.2	26.0 ± 3.0	27.4 ± 3.3	<0.001
WC, cm	94.8 ± 12.4	92.4 ± 11.3	97.3 ± 13.0	<0.001
Heart rate, bpm	71.7 ± 10.2	70.9 ± 9.3	72.5 ± 10.9	0.024
SBP, mmHg	131.8 ± 17.1	131.0 ± 16.7	132.6 ± 17.6	0.182
DBP, mmHg	76.8 ± 10.1	76.5 ± 9.9	77.1 ± 10.3	0.376
Smoking history, *n* (%)	417 (52.3)	218 (54.6)	199 (49.9)	0.178
Drinking history, *n* (%)	184 (23.1)	110 (27.6)	74 (18.5)	0.002
Family history of CAD, n (%)	93 (11.7)	38 (9.5)	55 (13.8)	0.061
Duration of diabetes, years	8.2 ± 4.3	8.4 ± 4.5	8.1 ± 4.1	0.220
Medical history, *n* (%)				
Hypertension	573 (71.8)	265 (66.4)	308 (77.2)	0.001
Previous MI	175 (21.9)	80 (20.1)	95 (23.8)	0.199
Previous PCI	151 (18.9)	76 (19.0)	75 (18.8)	0.928
Previous stroke	109 (13.7)	54 (13.5)	55 (13.8)	0.918
Previous PAD	125 (15.7)	64 (16.0)	61 (15.3)	0.770
Clinical diagnosis, *n* (%)				0.004
UA	650 (81.5)	341 (85.5)	309 (77.4)	
NSTEMI	148 (18.5)	58 (14.5)	90 (22.6)	
Laboratory examinations				
TG, mmol/L	1.6 (1.1, 2.2)	1.1 (0.9, 1.4)	2.2 (1.8, 3.0)	<0.001
TC, mmol/L	4.1 ± 1.0	3.9 ± 1.0	4.2 ± 1.0	0.001
LDL-C, mmol/L	2.4 ± 0.9	2.4 ± 0.8	2.5 ± 0.9	0.091
HDL-C, mmol/L	1.0 ± 0.2	1.1 ± 0.2	0.9 ± 0.2	<0.001
hs-CRP, mg/L	1.6 (0.7, 4.1)	1.2 (0.5, 3.6)	2.0 (1.0, 4.4)	<0.001
Creatinine, μmol/L	73.3 ± 16.9	74.3 ± 17.3	72.3 ± 16.4	0.090
eGFR, mL/(min ×1.73 m^2^)	96.5 ± 21.6	97.3 ± 21.4	95.8 ± 21.8	0.337
Uric acid, μmol/L	328.0 ± 75.6	318.7 ± 72.8	337.3 ± 77.2	0.001
FBG, mmol/L	7.7 ± 2.5	7.3 ± 2.3	8.0 ± 2.6	<0.001
HbA1c, %	7.5 ± 1.3	7.3 ± 1.3	7.6 ± 1.2	0.004
LVEF, %	64.0 ± 6.6	64.1 ± 6.6	63.8 ± 6.6	0.562

*BMI, body mass index; WC, waist circumference; SBP, systolic blood pressure; DBP, diastolic blood pressure; CAD, coronary artery disease; MI, myocardial infarction; PCI, percutaneous coronary intervention; PAD, peripheral artery disease; UA, unstable angina; NSTEMI, non-ST-segment elevation myocardial infarction; TG, triglyceride; TC, total cholesterol; LDL-C, low-density lipoprotein cholesterol; HDL-C, high-density lipoprotein cholesterol; hs-CRP, high-sensitivity C-reactive protein; eGFR, estimated glomerular filtration rate; FBG, fasting blood glucose; HbA1c, glycosylated hemoglobin A1c; LVEF, left ventricular ejection fraction; VAI, visceral adiposity index*.

**Table 2:** Therapeutic, angiographic, and procedural characteristics of the study population.

	**Total population (*n* = 798)**	**Lower VAI (<2.6; *n* = 399)**	**Higher VAI (≥2.6; *n* = 399)**	***P*-value**
Medication at admission, *n* (%)				
ACEI/ARB	207 (25.9)	84 (21.1)	123 (30.8)	0.002
DAPT	253 (31.7)	120 (30.1)	133 (33.3)	0.323
Aspirin	427 (53.5)	209 (52.4)	218 (54.6)	0.523
P2Y12 inhibitors	265 (33.2)	124 (31.1)	141 (35.3)	0.201
β-Blocker	166 (20.8)	71 (17.8)	95 (23.8)	0.036
Statins	233 (29.2)	121 (30.3)	112 (28.1)	0.483
OAD	413 (51.8)	198 (49.6)	215 (53.9)	0.228
Insulin	225 (28.2)	110 (27.6)	115 (28.8)	0.694
Medication at discharge, *n* (%)				
ACEI/ARB	618 (77.4)	282 (70.7)	336 (84.2)	<0.001
DAPT	797 (99.9)	398 (99.7)	399 (100.0)	>0.999
Aspirin	797 (99.9)	398 (99.7)	399 (100.0)	>0.999
P2Y12 inhibitors	798 (100.0)	399 (100.0)	399 (100.0)	-
β-Blocker	744 (93.2)	369 (92.5)	375 (94.0)	0.398
Statins	787 (98.6)	393 (98.5)	394 (98.7)	0.761
OAD	409 (51.3)	197 (49.4)	212 (53.1)	0.288
Insulin	217 (27.2)	104 (26.1)	113 (28.3)	0.474
Angiographic and procedural data				
LM disease, *n* (%)	44 (5.5)	21 (5.3)	23 (5.8)	0.756
Three-vessel disease, *n* (%)	344 (43.1)	176 (44.1)	168 (42.1)	0.567
In-stent restenosis, *n* (%)	58 (7.3)	29 (7.3)	29 (7.3)	-
SYNTAX score	12.0 ± 5.5	11.9 ± 5.2	12.2 ± 5.9	0.511
Target vessel, *n* (%)				
LM	25 (3.1)	14 (3.5)	11 (2.8)	0.542
LAD	513 (64.3)	253 (63.4)	260 (65.2)	0.605
LCX	335 (42.0)	178 (44.6)	157 (39.3)	0.132
RCA	398 (49.9)	193 (48.4)	205 (51.4)	0.396
Complete revascularization, *n* (%)	414 (51.9)	205 (51.4)	209 (52.4)	0.777
Number of DES	2.1 ± 1.3	2.1 ± 1.3	2.1 ± 1.3	0.912
TIMI score	3.2 ± 1.0	3.0 ± 1.0	3.3 ± 1.0	<0.001
GRACE score	76.4 ± 16.9	77.4 ± 15.4	75.3 ± 18.2	0.082

*ACEI, angiotensin-converting enzyme inhibitor; ARB, angiotensin receptor blocker; DAPT, dual antiplatelet therapy; OAD, oral antidiabetic drug; LM, left main; SYNTAX, synergy between PCI with taxus and cardiac surgery; LAD, left anterior descending artery; LCX, left circumflex; RCA, right coronary artery; DES, drug-eluting stent; TIMI, Thrombolysis in Myocardial Infarction; GRACE, Global Registry of Acute Coronary Events; VAI, visceral adiposity index*.

### Predictive Value of VAI for Adverse Prognosis

**Figure 1:**
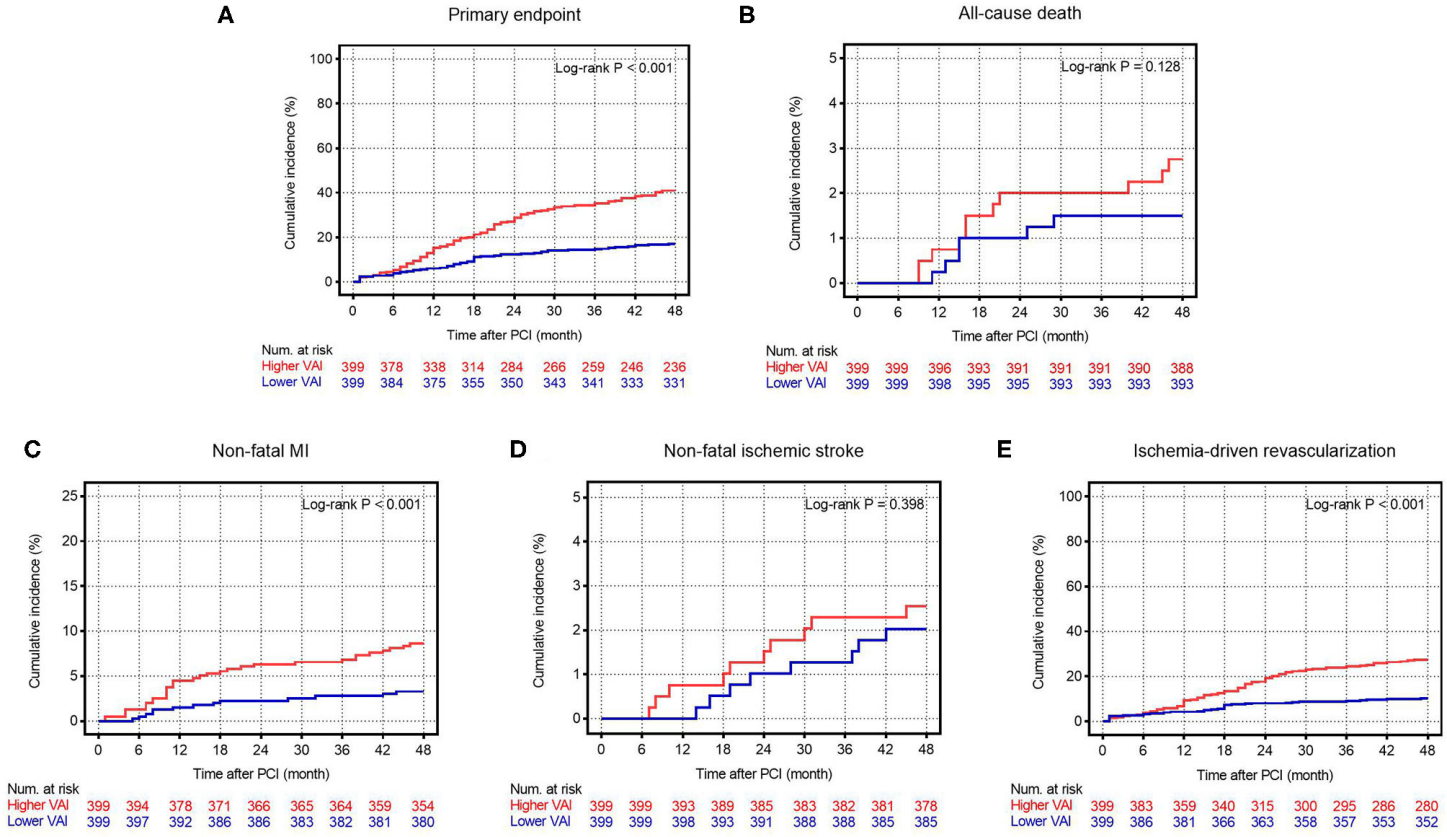
The Kaplan–Meier survival curves according to the median of VAI. **(A)** The Kaplan–Meier survival curves for the primary endpoint; **(B)** The Kaplan–Meier survival curves for all-cause death; **(C)** The Kaplan–Meier survival curves for non-fatal MI; **(D)** The Kaplan–Meier curves for non-fatal ischemic stroke; **(E)** The Kaplan–Meier curves for ischemia-driven revascularization. VAI, visceral adiposity index; PCI, percutaneous coronary intervention; MI, myocardial infarction.

Supplementary Table 1, which summarized the incidence of the primary endpoint and each component, showed that the incidence of the primary endpoint (*p* < 0.001), non-fatal MI (*p* = 0.002), and ischemia-driven revascularization (*p* < 0.001), but not all-cause death (*p* = 0.220) and non-fatal ischemic stroke (*p* = 0.633), increased significantly with the higher median of VAI. Similar results were obtained when evaluating the time-dependent cumulative incidence of the primary endpoint and each component between groups by using the Kaplan–Meier analysis (Figure 1).

In comparison with lower median, higher median of VAI was shown to be a significant predictor of the primary endpoint (unadjusted: HR 2.75, 95% CI 2.07–3.64, *p* < 0.001; fully adjusted model 5: HR 2.72, 95% CI 2.02–3.68, *p* < 0.001), non-fatal MI (unadjusted: HR 2.96, 95% CI 1.56–5.61, *p* < 0.001; fully adjusted model 5: HR 3.08, 95% CI 1.54–6.19, *p* = 0.002), and ischemia-driven revascularization (unadjusted: HR 3.01, 95% CI 2.10–4.31, *p* < 0.001; fully adjusted model 5: HR 3.10, 95% CI 2.11–4.54, *p* < 0.001), irrespective of the adjustment of confounding factors selected based on statistical significance in unadjusted analysis (*p* < 0.05, details shown in Supplementary Table 2) and clinical experience. In keeping with the primary analysis, the predictive value of VAI was further confirmed in sensitivity analysis where VAI was taken as a continuous variate. Of note, when being taken as a continuous variate, VAI was also revealed to be a risk predictor of all-cause death. However, VAI as a nominal variate failed to be associated with all-cause death, except for that adjusted for model 1. Detailed HR and 95% CI are shown in Tables 3, 4.

**Table 3:** Predictive value of VAI as a nominal variate for the primary endpoint and each component.

	**Primary endpoint**		**All-cause death**		**Non-fatal MI**		**Non-fatal ischemic stroke**		**Ischemia-driven revascularization**	
	**HR (95% CI)**	***P*-value**	**HR (95% CI)**	***P*-value**	**HR (95% CI)**	***P*-value**	**HR (95% CI)**	***P*-value**	**HR (95% CI)**	***P*-value**
Unadjusted	2.75 (2.07–3.64)	<0.001	2.13 (0.79–5.76)	0.137	2.96 (1.56–5.61)	0.001	1.49 (0.59–3.78)	0.401	3.01 (2.10–4.31)	<0.001
Model 1	3.09 (2.31–4.15)	<0.001	2.94 (1.04–8.32)	0.042	3.01 (1.55–5.84)	0.001	1.27 (0.48–3.37)	0.633	3.50 (2.42–5.08)	<0.001
Model 2	2.98 (2.21–4.01)	<0.001	2.25 (0.76–6.71)	0.145	3.13 (1.58–6.20)	0.001	1.27 (0.47–3.46)	0.641	3.37 (2.32–4.91)	<0.001
Model 3	2.77 (2.05–3.73)	<0.001	2.13 (0.67–6.71)	0.198	2.98 (1.49–5.95)	0.002	1.30 (0.47–3.63)	0.615	3.14 (2.15–4.58)	<0.001
Model 4	2.75 (2.04–3.71)	<0.001	2.21 (0.65–7.47)	0.203	2.94 (1.47–5.88)	0.002	1.20 (0.43–3.38)	0.725	3.11 (2.13–4.55)	<0.001
Model 5	2.72 (2.02–3.68)	<0.001	1.78 (0.49–6.50)	0.383	3.08 (1.54–6.19)	0.002	1.21 (0.42–3.47)	0.721	3.10 (2.11–4.54)	<0.001

*Model 1: Adjusted for age and gender*.

*Model 2: Adjusted for variates in model 1 and SBP, DBP, smoking history, duration of diabetes, previous MI, previous PCI, previous stroke, and diagnosis*.

*Model 3: Adjusted for variates in model 2 and TC, eGFR, HbA1c, and LVEF*.

*Model 4: Adjusted for variates in model 3 and statins at admission, OAD at admission, insulin at admission, and ACEI/ARB at discharge*.

*Model 5: Adjusted for variates in model 4 and the SYNTAX score, LM treatment, complete revascularization, and number of DES*.

*The HR was evaluated regarding the lower median of VAI as reference*.

*SBP, systolic blood pressure; DBP, diastolic blood pressure; MI myocardial infarction, PCI, percutaneous coronary intervention; OAD, oral antiplatelet drug; eGFR, estimated glomerular filtration rate; HbA1c, glycosylated hemoglobin A1c; TC, total cholesterol; ACEI, angiotensin-converting enzyme inhibitor; ARB, angiotensin receptor blocker; LM, left main; DES, drug-eluting stent; LVEF, left ventricular ejection fraction; VAI, visceral adiposity index; SYNTAX, synergy between PCI with taxus and cardiac surgery; HR hazard ratio*.

**Table 4:** Predictive value of VAI as a continuous variate for the primary endpoint and each component.

	**Primary endpoint**		**All-cause death**		**Non-fatal MI**		**Non-fatal ischemic stroke**		**Ischemia-driven revascularization**	
	**HR (95% CI)**	***P*-value**	**HR (95% CI)**	***P*-value**	**HR (95% CI)**	***P*-value**	**HR (95% CI)**	***P*-value**	**HR (95% CI)**	***P*-value**
Unadjusted	1.29 (1.24–1.35)	<0.001	1.23 (1.04–1.45)	0.018	1.37 (1.26–1.49)	<0.001	1.06 (0.85–1.31)	0.635	1.29 (1.23–1.36)	<0.001
Model 1	1.34 (1.28–1.40)	<0.001	1.34 (1.12–1.61)	0.002	1.40 (1.27–1.53)	<0.001	1.00 (0.79–1.28)	0.977	1.35 (1.28–1.43)	<0.001
Model 2	1.34 (1.28–1.40)	<0.001	1.33 (1.09–1.63)	0.005	1.39 (1.27–1.53)	<0.001	0.99 (0.77–1.28)	0.954	1.34 (1.27–1.42)	<0.001
Model 3	1.34 (1.27–1.40)	<0.001	1.36 (1.09–1.71)	0.006	1.45 (1.31–1.61)	<0.001	0.98 (0.76–1.27)	0.881	1.34 (1.26–1.42)	<0.001
Model 4	1.33 (1.27–1.40)	<0.001	1.41 (1.12–1.79)	0.004	1.46 (1.32–1.62)	<0.001	0.97 (0.75–1.27)	0.848	1.33 (1.26–1.41)	<0.001
Model 5	1.34 (1.28–1.41)	<0.001	1.38 (1.06–1.79)	0.017	1.49 (1.34–1.67)	<0.001	0.98 (0.75–1.29)	0.876	1.34 (1.26–1.42)	<0.001

*Model 1: Adjusted for age and gender*.

*Model 2: Adjusted for variates in model 1 and SBP, DBP, smoking history, duration of diabetes, previous MI, previous PCI, previous stroke, and diagnosis*.

*Model 3: Adjusted for variates in model 2 and TC, eGFR, HbA1c, and LVEF*.

*Model 4: Adjusted for variates in model 3 and statins at admission, OAD at admission, insulin at admission, and ACEI/ARB at discharge*.

*Model 5: Adjusted for variates in model 4 and the SYNTAX score, LM treatment, complete revascularization, and number of DES*.

*The HR was evaluated by per 1-unit increase of VAI*.

*MI, myocardial infarction; HR, hazard ratio*.

**Figure 2:**
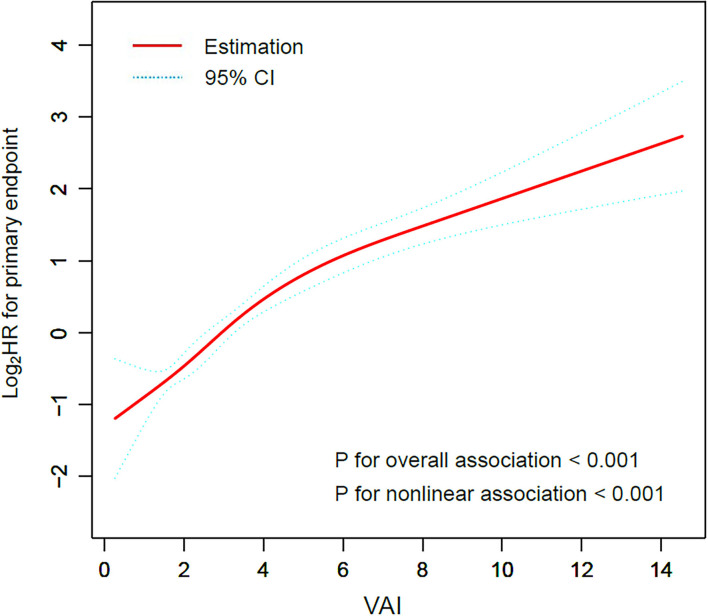
Restricted cubic smoothing for the risk of the primary endpoint according to the VAI. The analysis was adjusted for model 5. HR was evaluated by per 1-unit increase of VAI. VAI, visceral adiposity index; HR, hazard ratio.

Restricted cubic smoothing was performed to elucidate the potential dose-response relationship between VAI and the risk of the primary endpoint with the adjustment for model 5 (Figure 2). The smoothing curve showed that the risk of the primary endpoint ascended with the increase of VAI (*p* for overall association < 0.001), indicating a linear relationship between VAI and the risk of the primary endpoint, which was further confirmed by the test of non-linear association (*p* for non-linear association < 0.001).

**Figure 3:**
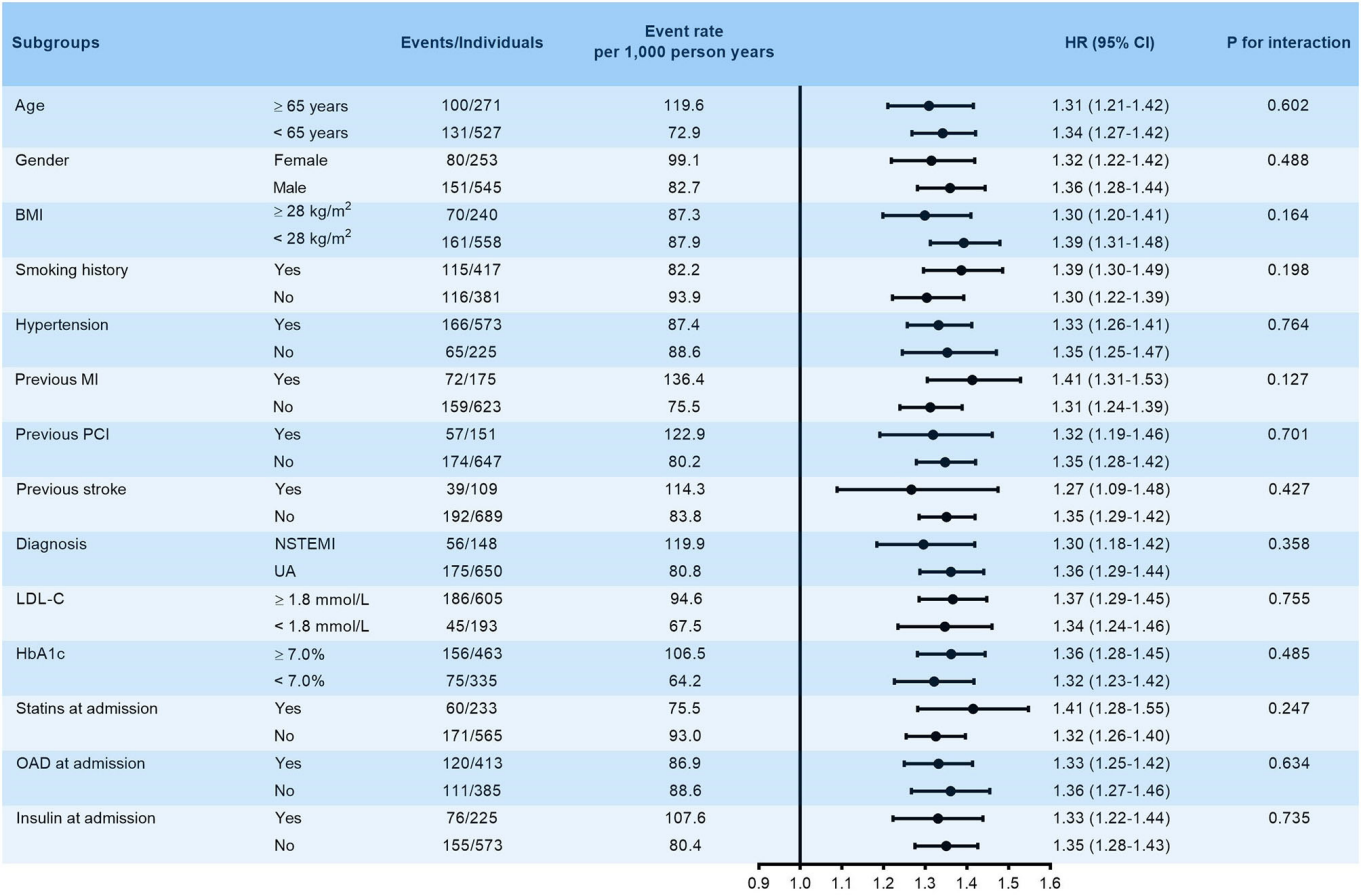
Subgroup analysis evaluating the robustness of VAI in predicting the risk of the primary endpoint. The analysis was adjusted for model 5 except for variates applied for grouping. HR was evaluated by per 1-unit increase of VAI. BMI, body mass index; MI, myocardial infarction; PCI, percutaneous coronary intervention; NSTEMI, non-ST-segment elevation myocardial infarction; UA, unstable angina; LDL-C, low-density lipoprotein cholesterol; HbA1c, glycosylated hemoglobin A1c; HR, hazard ratio.

The robustness of VAI in predicting the primary endpoint was subsequently assessed by subgroup analysis. VAI was stubbornly shown to be a significant predictor of the primary endpoint in various subgroups stratified by age (≥ 65 or <65 years), gender (female or male), BMI (≥ 28 or <28 kg/m^2^), smoking history (yes or no), hypertension (yes or no), previous history of MI, PCI and stroke (yes or no), diagnosis (NSTEMI or UA), LDL-C (≥ 1.8 or <1.8 mmol/L), HbA1c (≥ 7.0 or <7.0%), and treatment at admission including statins, OAD, and insulin (yes or no) (all *p* for interaction > 0.05, Figure 3).

### Comparison of Prognostic Impact Between VAI and Existing Risk Scores

Compared with the TIMI score (Harrell's C-index: VAI vs. TIMI score, 0.694 vs. 0.643, *p* for comparison = 0.073; continuous NRI: −0.019, *p* = 0.618; IDI: −0.047, *p* = 0.080) and the GRACE score (Harrell's C-index: VAI vs. GRACE score, 0.694 vs. 0.640, *p* for comparison = 0.075; continuous NRI: −0.083, *p* = 0.179; IDI: −0.037, *p* = 0.246), VAI did not exhibit a stronger ability on risk stratification for the primary endpoint (Table 5).

**Table 5:** Comparison of VAI with existing risk scores on risk stratification for the primary endpoint.

	**Harrell's C-index**			**Continuous-NRI**			**IDI**		
	**Estimation**	**95% CI**	***P* for comparison**	**Estimation**	**95% CI**	***P*-value**	**Estimation**	**95% CI**	***P*-value**
VAI	0.694	0.658–0.729	-	-	-	-	-	-	-
TIMI score	0.643	0.609–0.676	0.073	−0.019	−0.190–0.106	0.618	−0.047	−0.103–0.003	0.080
GRACE score	0.640	0.602–0.679	0.075	−0.083	−0.222–0.040	0.179	−0.037	−0.103–0.026	0.246

*VAI, visceral adiposity index; TIMI, the Thrombolysis in Myocardial Infarction; GRACE, the Global Registry of Acute Coronary Events; NRI, net reclassification improvement; IDI, integrated discrimination improvement*.

On the basis of the TIMI score and the GRACE score, the addition of VAI displayed significant incremental effects on risk stratification for the primary endpoint, expressed as increased Harrell's C-index (TIMI score vs. + VAI: 0.643 vs. 0.736, *p* for comparison < 0.001; GRACE score vs. + VAI: 0.640 vs. 0.750, *p* for comparison < 0.001), significant continuous-NRI (TIMI score vs. + VAI: 0.349, *p* < 0.001; GRACE score vs. + VAI: 0.382, *p* < 0.001), and significant IDI (TIMI score vs. + VAI: 0.113, *p* < 0.001; GRACE score vs. + VAI: 0.135, *p* < 0.001). Details are shown in Table 6.

**Table 6:** Incremental effects of VAI on risk stratification for the primary endpoint beyond existing risk scores.

	**Harrell's C-index**			**Continuous-NRI**			**IDI**		
	**Estimation**	**95% CI**	***P* for comparison**	**Estimation**	**95% CI**	***P*-value**	**Estimation**	**95% CI**	***P*-value**
TIMI score	0.643	0.609–0.676	-	-	-	-	-	-	-
+ VAI	0.736	0.704–0.767	<0.001	0.349	0.275–0.412	<0.001	0.113	0.080–0.150	<0.001
GRACE score	0.640	0.602–0.679	-	-	-	-	-	-	-
+ VAI	0.750	0.718–0.782	<0.001	0.382	0.292–0.451	<0.001	0.135	0.098–0.179	<0.001

*TIMI, the Thrombolysis in Myocardial Infarction; VAI, visceral adiposity index; GRACE, the Global Registry of Acute Coronary Events; NRI, net reclassification improvement; IDI, integrated discrimination improvement*.

## Discussion

This study showed that the elevated level of VAI is significantly related to the increased risk of adverse cardiovascular outcomes in the study population with the adjustment for potential confounding factors. VAI exhibited a significant incremental effect on risk stratification for adverse prognosis on the basis of existing risk scores. To the best of our knowledge, this is the first study investigating the potential of VAI in predicting adverse outcomes after the treatment of elective PCI in a selected high-risk cohort of NSTE-ACS accompanied with T2DM.

As one of the common risk factors, T2DM has been generally proved to be closely associated with the occurrence, progression, and deterioration of cardiovascular disease (4, 5). Study has shown that the close relationship of T2DM with cardiovascular disease is mainly mediated by IR (29), which is the most important mechanism of T2DM and metabolic syndrome (MetS), and characterized by decreasing efficiency of insulin in promoting glucose utilization and the compensatory secretion of more insulin producing hyperinsulinemia to maintain glycometabolic stability (30). Therefore, it appeals to great necessities on quantification of the extent of IR in patients with T2DM who are susceptible to or have experienced cardiovascular disease with the aim to improve the process of risk stratification and prognostic prediction. The gold standard technique for evaluating IR has been recognized as HIEG clamp, but the defects of operational complexity and expensiveness confined it from extensive clinical application. Former studies showed that IR usually manifested as increased fasting glucose, hyperinsulinemia, hypertriglyceridemia, decreased HDL-C, and obesity (especially increased visceral fat) (8). Referring to these characteristics, various indices calculated from common anthropometric and laboratory parameters (e.g., fasting glucose, insulin, TG, HDL-C, WC, BMI, etc.) have been proposed to alternatively evaluate the extent of IR (31). Among the parameters mentioned above, however, the levels of fasting glucose and insulin may be significantly affected by antidiabetic treatments, especially for individuals with diabetes. Meanwhile, the heterogeneity between different laboratories on the measurement of insulin is ubiquitous. Thus, VAI, determined by WC, BMI, fasting TG, and HDL-C, was established and thought to be a more comprehensive and less-affected indicator of IR (22). Unlike the gold standard HIEG clamp and other surrogate markers of IR, which are complicated, time- and cost-consuming, and glucose and insulin dependent, VAI exhibited the superiority of simplicity, accessibility, inexpensiveness, and glucose and insulin independent. Studies have confirmed the significant correlation between VAI and HIEG clamp and the HOMA-IR (9, 10), suggesting the great potential of VAI as a useful indicator to accurately reflect the level of IR.

Studies have shown that VAI is significantly associated with the prevalence of T2DM (32, 33), prediabetes particularly impaired fasting glucose (34, 35) and MetS (36), independent of the components of VAI and other confounding variates. These findings suggested that VAI is an important indicator for assessing the incidence of T2DM, prediabetes, and MetS and, therefore, a useful tool for early identification of individuals who are prone to developing these disorders. However, VAI performs better than common anthropometric indicators in predicting abnormal glucose metabolisms and MetS remains controversial (37–44). Studies also showed that VAI is significantly associated with the coronary artery calcium score (11) and carotid intima-media thickness (12), both of which are well-recognized risk factors for atherosclerosis, indicating that VAI is useful for identifying the patients with high susceptibility for cardiovascular disease. The association between VAI and the incidence and complexity of cardiovascular disease has also been demonstrated by certain researches (13–19). A study has shown that VAI is not superior to common anthropometric measures in predicting the risk of cardiovascular disease (45). When investigating the impact of VAI on prognostic prediction in patients with preexisting cardiovascular disease, nevertheless, VAI was not shown as a significant risk predictor for adverse prognosis (16, 46).

This study, which identified the significant prognostic impact of VAI in a specific high-risk group with NSTE-ACS and T2DM undergoing elective PCI, is an important exploration and supplement to previous studies. Results from multivariate and subgroup analyses aimed at eliminating the influences of confounding factors showed that VAI was significantly and consistently associated with worse outcomes, indicating the robustness of VAI as a simple surrogate of IR in predicting the risk of adverse prognosis. Although VAI did not show a stronger value of risk stratification than the TIMI score and the GRACE score, the addition of VAI to these risk scores displayed a significant incremental effect on risk stratification, suggesting that VAI may provide additional information in risk prediction and stratification on the basis of existing risk scores.

The close relationship between VAI and worse prognosis may be mediated by IR, which promotes the formation and development of atherosclerosis through various mechanisms. IR can facilitate the phosphorylation of transcription factors through the mitogen-activated protein kinase (MAPK) pathway, thus promoting the proliferation and differentiation of vascular smooth muscle cells and activating the inflammatory reaction, which may, in turn, further aggravate the degree of IR and then leads to a vicious circle (7, 47). Studies revealed that IR plays an important role in the activation of nitric oxide (NO), a powerful vascular endothelial regulator, dysregulation of which can cause vascular endothelial dysfunction. This may be the most important mechanism linking IR and cardiovascular disease at the cellular level (47, 48). Studies have also shown that IR stimulates the production of endothelin-1 and then further promotes the increasing of vasoconstrictive tension and the progression of atherosclerosis (49). It has been demonstrated that IR is also related to oxidative stress, cardiovascular remodeling, incomplete myocardial perfusion, impaired microcirculatory function, and coagulation imbalance, all of which have a significant impact on the development of cardiovascular disease (50–52). Overall, since this study is hypothesis generating, further studies are required to investigate the potential pathophysiological process and mechanism inducing the relationship of VAI with cardiovascular disease.

Despite the extensive implementation of optimized therapies, the incidence of recurrent adverse events remains comparatively high for those with cardiovascular disease, particularly for high-risk groups like the one in this study. There is an urgent need to develop novel therapeutic targets to optimize treatments and improve prognosis ulteriorly. Studies targeting whether interventions on IR assessed by surrogate markers have a favorable effect on the prognosis remain relatively scarce at present. Former studies showed a significant association of the Dietary Approaches to Stop Hypertension (DASH) diet pattern (53), consumption of extra-virgin olive oil (54) with decreasing level of VAI, suggesting that well lifestyles play important effects on alleviating visceral adiposity, thus relieving the extent of IR. In addition, empagliflozin (55) and liraglutide (56) were proved to have a positive effect on regulating the level of VAI. Study has also confirmed that the level of microRNA 33a and 33b was positively correlated with VAI, indicating gene therapies such as small interfering RNA may be promising strategies (57). Future interventional studies are needed to investigate whether management on IR evaluated by VAI improves clinical prognosis in patients with cardiovascular disease.

Some limitations listed as follows need to be noted. Firstly, the design of single-center, retrospective cohort study and the relatively small sample capacity may weaken the statistical power. Secondly, despite multiple monitors of VAI during the follow-up may provide more convincing results, it was not accessible in this study. Thirdly, the lipid-lowering and antidiabetic therapy at admission, though adjusted or alleviated in analysis, may have an underlying impact on study results. Fourthly, the gold standard and generally accepted methods evaluating IR, HIEG clamp, and the HOMA-IR were unattainable in this study, which makes the comparison between VAI and them unavailable. Additionally, the use of some types of OAD such as thiazolidinediones may reduce TG levels and then affect the data of VAI. However, the detailed classification of OAD was not accessible in this study. Finally, patients with in-hospital death were excluded in the current analysis, which makes it unable to evaluate the predictive value of VAI in in-hospital adverse outcomes. Further studies are required to answer this question.

## Conclusion

Visceral adiposity index is significantly related to the risk of adverse prognosis in this selected population with NSTE-ACS and T2DM receiving elective PCI. Significant incremental effects on risk stratification for adverse prognosis are obtained after the addition of VAI to existing risk scores. These findings indicate that VAI can be served as a useful tool for risk stratification in this specific population. The present results require further large-scale, prospective studies to confirm.

## Data Availability Statement

The raw data supporting the conclusions of this article will be made available by the authors, without undue reservation.

## Ethics Statement

The studies involving human participants were reviewed and approved by the Clinical Research Ethics Committee of Beijing Anzhen Hospital, Capital Medical University. The patients/participants provided their written informed consent to participate in this study.

## Author Contributions

QZ made substantial contributions to study design, data collection, follow-up, data analysis, and manuscript writing. Y-JZ made substantial contributions to study design and intellectual direction. Y-JC, Y-KX, Z-WZ, CL, and T-NS made contributions to data collection and analysis. All the authors read and approved the final manuscript.

## Funding

This study was supported by the grant from National Key Research and Development Program of China (2017YFC0908800), the Beijing Municipal Administration of Hospitals Mission Plan (SML20180601), the Capital's Funds for Health Improvement and Research (CFH2020-2-2063) (KM200910025012), and the Beijing Municipal Natural Science Foundation (7202041).

## Conflict of Interest

The authors declare that the research was conducted in the absence of any commercial or financial relationships that could be construed as a potential conflict of interest.

## Publisher's Note

All claims expressed in this article are solely those of the authors and do not necessarily represent those of their affiliated organizations, or those of the publisher, the editors and the reviewers. Any product that may be evaluated in this article, or claim that may be made by its manufacturer, is not guaranteed or endorsed by the publisher.

## Supplementary Material

Supplementary Figure 1 Flow diagram for the enrollment of study population. NSTE-ACS, non-ST-segment elevation acute coronary syndrome; PCI, percutaneous coronary intervention; T1DM, type 1 diabetes mellitus; CABG, coronary artery bypass grafting; TG, triglyceride; eGFR, estimated glomerular filtration rate; ALT, alanine transaminase; AST, aspartate transaminase; URL, upper reference limit; BMI, body mass index; MI, myocardial infarction.

Supplementary Table 1 Incidence of primary endpoint and each component according to the median of VAI. MI, myocardial infarction; VAI, visceral adiposity index.

Supplementary Table 2 Unadjusted Cox regression analysis investigating predictors of primary endpoint. BMI, body mass index; WC, waist circumference; SBP, systolic blood pressure; DBP, diastolic blood pressure; CAD, coronary artery disease; MI, myocardial infarction; PCI, percutaneous coronary intervention; PAD, peripheral artery disease; UA, unstable angina; TG, triglyceride; TC, total cholesterol; LDL-C, low-density lipoprotein cholesterol; HDL-C, high-density lipoprotein cholesterol; hs-CRP, high-sensitivity C-reactive protein; eGFR, estimated glomerular filtration rate; FBG, fasting blood glucose; HbA1c, glycosylated hemoglobin A1c; LVEF, left ventricular ejection fraction; ACEI, angiotensin converting enzyme inhibitor; ARB, angiotensin receptor blocker; DAPT, dual antiplatelet therapy; OAD, oral antidiabetic drugs; LM, left main artery; SYNTAX, synergy between PCI with taxus and cardiac surgery; LAD, left anterior descending artery; LCX, left circumflex artery; RCA, right coronary artery; DES, drug-eluting stent.
