# Supplementary figures and images for: Visceral Adiposity Index Plays an Important Role in Prognostic Prediction in Patients With Non-ST-Segment Elevation Acute Coronary Syndrome and Type 2 Diabetes Mellitus Undergoing Percutaneous Coronary Intervention
Source: Front Cardiovasc Med. 2021 Nov 18;8:735637. doi: 10.3389/fcvm.2021.735637 (PMC8636737; doi:10.3389/fcvm.2021.735637)

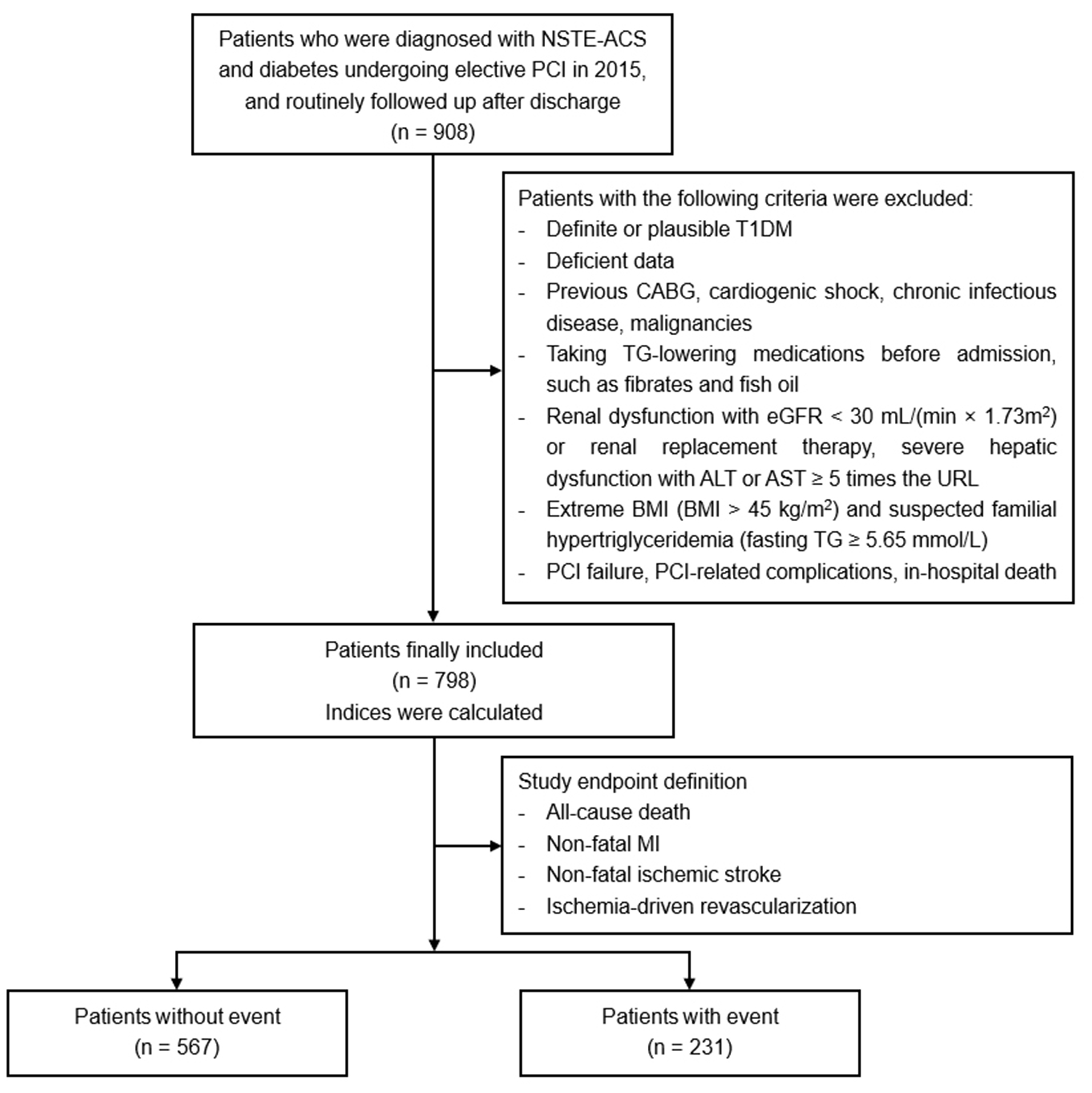

Supplement: Supplementary Figure 1 — Flow diagram for the enrollment of study population. NSTE-ACS, non-ST-segment elevation acute coronary syndrome; PCI, percutaneous coronary intervention; T1DM, type 1 diabetes mellitus; CABG, coronary artery bypass grafting; TG, triglyceride; eGFR, estimated glomerular filtration rate; ALT, alanine transaminase; AST, aspartate transaminase; URL, upper reference limit; BMI, body mass index; MI, myocardial infarction. [file Image_1.JPEG]
