# Supplementary material for: Visceral Adiposity Index Plays an Important Role in Prognostic Prediction in Patients With Non-ST-Segment Elevation Acute Coronary Syndrome and Type 2 Diabetes Mellitus Undergoing Percutaneous Coronary Intervention
Source: Front Cardiovasc Med. 2021 Nov 18;8:735637. doi: 10.3389/fcvm.2021.735637 (PMC8636737; doi:10.3389/fcvm.2021.735637)
Supplement: Supplementary Table 1 — Incidence of primary endpoint and each component according to the median of VAI. MI, myocardial infarction; VAI, visceral adiposity index. [file Table_1.DOCX]

## Table S1. Incidence of primary endpoint and each component according to the median of VAI

|  | Total population  (n = 798) | Lower VAI  (< 2.6; n = 399) | Higher VAI  (≥ 2.6; n = 399) | *P* value |
| --- | --- | --- | --- | --- |
| Primary endpoint, n (%) | 231 (28.9) | 68 (17.0) | 163 (40.9) | < 0.001 |
| All-cause death, n (%) | 17 (2.1) | 6 (1.5) | 11 (2.8) | 0.220 |
| Non-fatal MI, n (%) | 47 (5.9) | 13 (3.3) | 34 (8.5) | 0.002 |
| Non-fatal ischemic stroke, n (%) | 18 (2.3) | 8 (2.0) | 10 (2.5) | 0.633 |
| Ischemia-driven revascularization, n (%) | 149 (18.7) | 41 (10.3) | 108 (27.1) | < 0.001 |

*MI* myocardial infarction, *VAI* visceral adiposity index

## Table S2. Unadjusted Cox regression analysis investigating predictors of primary endpoint

|  | Primary endpoint | | |
| --- | --- | --- | --- |
|  | HR | 95% CI | *P* value |
| Age, per 1 years | 1.02 | 1.00-1.04 | 0.012 |
| Gender, male as reference | 1.19 | 0.91-1.56 | 0.204 |
| BMI, per 1 kg/m^2^ | 1.01 | 0.97-1.06 | 0.495 |
| WC, per 1 cm | 1.03 | 1.02-1.04 | < 0.001 |
| Heart rate, per 1 bpm | 1.01 | 1.00-1.02 | 0.137 |
| SBP, per 1 mmHg | 1.01 | 1.00-1.02 | 0.006 |
| DBP, per 1 mmHg | 1.01 | 1.00-1.03 | 0.030 |
| Smoking history | 0.88 | 0.68-1.14 | 0.328 |
| Drinking history | 0.88 | 0.64-1.20 | 0.421 |
| Family history of CAD | 0.94 | 0.62-1.41 | 0.762 |
| Duration of diabetes, per 1 year | 1.04 | 1.01-1.07 | 0.018 |
| Hypertension | 0.99 | 0.74-1.31 | 0.926 |
| Previous MI | 1.78 | 1.34-2.35 | < 0.001 |
| Previous PCI | 1.51 | 1.12-2.04 | 0.007 |
| Previous stroke | 1.35 | 0.95-1.90 | 0.092 |
| Previous PAD | 1.25 | 0.89-1.74 | 0.193 |
| Diagnosis, UA as reference | 1.47 | 1.09-1.99 | 0.012 |
| TG, per 1 mmol/L | 1.75 | 1.58-1.95 | < 0.001 |
| TC, per 1 mmol/L | 1.20 | 1.07-1.35 | 0.001 |
| LDL-C, per 1 mmol/L | 1.05 | 0.91-1.22 | 0.479 |
| HDL-C, per 1 mmol/L | 0.17 | 0.09-0.34 | < 0.001 |
| hs-CRP, per 1 mg/L | 1.01 | 1.00-1.03 | 0.167 |
| eGFR, per 1 mL/(min × 1.73m^2^) | 0.99 | 0.99-1.00 | 0.053 |
| FBG, per 1 mmol/L | 1.09 | 1.05-1.14 | < 0.001 |
| HbA1c, per 1% | 1.26 | 1.15-1.37 | < 0.001 |
| LVEF, per 1% | 0.97 | 0.95-0.99 | 0.003 |
| ACEI/ARB at admission | 1.14 | 0.86-1.51 | 0.374 |
| DAPT at admission | 1.08 | 0.82-1.43 | 0.564 |
| Statins at admission | 0.82 | 0.61-1.09 | 0.173 |
| OAD at admission | 0.98 | 0.76-1.27 | 0.864 |
| Insulin at admission | 1.32 | 1.01-1.74 | 0.045 |
| ACEI/ARB at discharge | 1.63 | 1.15-2.32 | 0.007 |
| Statins at discharge | 0.70 | 0.26-1.87 | 0.475 |
| OAD at discharge | 0.96 | 0.74-1.25 | 0.768 |
| Insulin at discharge | 1.34 | 1.02-1.76 | 0.039 |
| SYNTAX score, per 1-point | 1.11 | 1.08-1.13 | < 0.001 |
| LM treatment | 2.23 | 1.27-3.89 | 0.005 |
| LAD treatment | 1.18 | 0.90-1.56 | 0.231 |
| LCX treatment | 1.23 | 0.95-1.59 | 0.120 |
| RCA treatment | 1.27 | 0.98-1.64 | 0.076 |
| Complete revascularization | 0.80 | 0.62-1.04 | 0.095 |
| Number of DES, per 1 DES | 1.20 | 1.10-1.31 | < 0.001 |

*BMI* body mass index, *WC* waist circumference, *SBP* systolic blood pressure, *DBP* diastolic blood pressure, *CAD* coronary artery disease, *MI* myocardial infarction, *PCI* percutaneous coronary intervention, *PAD* peripheral artery disease, *UA* unstable angina, *TG* triglyceride, *TC* total cholesterol, *LDL-C* low-density lipoprotein cholesterol, *HDL-C* high-density lipoprotein cholesterol, *hs-CRP* high-sensitivity C-reactive protein, *eGFR* estimated glomerular filtration rate, *FBG* fasting blood glucose, *HbA1c* glycosylated hemoglobin A1c, *LVEF* left ventricular ejection fraction, *ACEI* angiotensin converting enzyme inhibitor, *ARB* angiotensin receptor blocker, *DAPT* dual antiplatelet therapy, *OAD* oral antidiabetic drugs, *LM* left main artery, *SYNTAX* synergy between PCI with taxus and cardiac surgery, *LAD* left anterior descending artery, *LCX* left circumflex artery, *RCA* right coronary artery, *DES* drug-eluting stent
